# Identification of methylation sites and signature genes with prognostic value for luminal breast cancer

**DOI:** 10.1186/s12885-018-4314-9

**Published:** 2018-04-11

**Authors:** Bin Xiao, Lidan Chen, Yongli Ke, Jianfeng Hang, Ling Cao, Rong Zhang, Weiyun Zhang, Yang Liao, Yang Gao, Jianyun Chen, Li Li, Wenbo Hao, Zhaohui Sun, Linhai Li

**Affiliations:** 1 Department of Laboratory Medicine, General Hospital of Guangzhou Military Command of PLA, Guangzhou, 510010 Guangdong China; 2 Department of Breast Surgery, General Hospital of Guangzhou Military Command of PLA, Guangzhou, 510010 Guangdong China; 3 Institute of Antibody Engineering, School of Biotechnology, Southern Medical University, Guangzhou, China; 4 State Key Laboratory of Organ Failure, Institute of Antibody Engineering, School of Biotechnology, Southern Medical University, Guangzhou, China; 5 Guangdong Provincial Key Laboratory of Tropical Disease Research, School of Public Health, Southern Medical University, Guangzhou, China

**Keywords:** Luminal breast cancer, Methylation, mRNA, Prognosis, *SOSTDC1*

## Abstract

**Background:**

Robust and precise molecular prognostic predictors for luminal breast cancer are required. This study aimed to identify key methylation sites in luminal breast cancer, as well as precise molecular tools for predicting prognosis.

**Methods:**

We compared methylation levels of normal and luminal breast cancer samples from The Cancer Genome Atlas dataset. The relationships among differentially methylated sites, corresponding mRNA expression levels and prognosis were further analysed. Differentially expressed genes in normal and cancerous samples were analysed, followed by the identification of prognostic signature genes. Samples were divided into low- and high-risk groups based on the signature genes. Prognoses of low- and high-risk groups were compared. The Gene Expression Omnibus dataset were used to validate signature genes for prognosis prediction. Prognosis of low- and high-risk groups in Luminal A and Luminal B samples from the TCGA and the Metabric cohort dataset were analyzed. We also analysed the correlation between clinical features of low- and high- risk groups as well as their differences in gene expression.

**Results:**

Fourteen methylation sites were considered to be related to luminal breast cancer prognosis because their methylation levels, mRNA expression and prognoses were closely related to each other. The methylation level of *SOSTDC1* was used to divide samples into hypo- and hyper-methylation groups. We also identified an mRNA signature, comprising eight transcripts, *ESCO2*, *PACSIN1*, *CDCA2*, *PIGR*, *PTN*, *RGMA*, *KLK4* and *CENPA*, which was used to divide samples into low- and high-risk groups. The low-risk group showed significantly better prognosis than the high-risk group. A correlation analysis revealed that the risk score was an independent prognostic factor. Low- and high- risk groups significantly correlated with the survival ratio in Luminal A samples, but not in Luminal B samples on the basis of the TCGA and the Metabric cohort dataset. Further functional annotation demonstrated that the differentially expressed genes were mainly involved in cell cycle and cancer progression.

**Conclusions:**

We identified several key methylation sites and an mRNA signature for predicting luminal breast cancer prognosis. The signature exhibited effective and precise prediction of prognosis and may serve as a prognostic and diagnostic marker for luminal breast cancer.

**Electronic supplementary material:**

The online version of this article (10.1186/s12885-018-4314-9) contains supplementary material, which is available to authorized users.

## Background

Breast cancer is one of the most commonly diagnosed cancers and one of the leading causes of death among female cancer patients. It has been estimated that, globally, approximately 12% of the newly diagnosed breast cancers occur in China [1]. Despite great efforts spent on improving the diagnosis and treatment of breast cancer, its prognosis varies greatly among patients. An effective molecular tool is urgently needed for predicting and classifying prognoses of breast cancer patients [2].

Cancer is caused by the accumulation of mutations in cancer susceptibility genes and the resulting abnormal cell growth. In addition to genetic variations, aberrant DNA methylation and variations in gene expression patterns have also been recognised to play an important role in tumourigenesis [3, 4]. Extensive studies have shown that global DNA hypomethylation and regional hypermethylation of Cytosine-Phosphate-Guanine (CpG) -rich islands are prevalent in cancers [4, 5]. Promoter methylation suppresses gene transcription, and aberrant methylation is one of the major causes leading to instability of the genome, activation of oncogenes and suppression of tumour suppressor genes. Accordingly, aberrant methylation may contribute greatly to breast cancer onset and progression.

Based on variations in gene expression, breast cancer is currently classified into five major subtypes: luminal A, luminal B, ErbB2+, basal-like and normal-like. However, based on the copy number, gene expression and long-term clinical outcomes, breast cancer is further divided into at least 10 intrinsic subtypes, which demonstrate the complexity of the landscape of breast cancer [6]. Each subtype has a unique expression pattern and unique clinical features [3, 7] and has a distinct response profile to the same therapy [8]. Thus, attempts to define the prognosis related gene expression signature remain necessary.

Specific methylation profiles may also exist for different subtypes. Holm et al. [9] have reported that certain patterns of hypermethylation, which modulate gene expression and promote tumor progression, may be viable targets in some luminal breast cancers. Reportedly, CpGs in the luminal B subtype are the most frequently methylated and those in the basal-like subtype are the least frequently methylated [10]. Significantly higher methylation levels of tumour suppressor genes Ras Association Domain family 1 (*RASSF1*) and Glutathione S-transferase Pi 1 (*GSTP1*) have been observed in the luminal B subtype than in the basal-like subtype [10]. Furthermore, the expression levels of both genes have been shown to be downregulated by hypermethylation in breast cancer [11–15]. The hypermethylation and reduced expression of *RASSF1* and *GSTP1* have been correlated with cancer onset and progression [13, 14].

Despite extensive investigations into aberrant methylation and gene expression, robust and precise molecular prognostic predictors for specific breast cancer subtypes, such as luminal A and B types, remain to be developed. In the present study, we used the data from The Cancer Genome Atlas (TCGA) as a training set and identified methylation sites that are significantly correlated with luminal breast cancer prognosis. The mRNA expression of genes corresponding to these sites correlated significantly with their methylation levels and prognoses. We further compared mRNA expression profiles between breast cancer and normal tissues and identified eight signature genes used for constructing a risk scoring system. Based on this system, luminal breast cancer patients were classified into low-risk and high-risk groups, which exhibited significant prognostic and molecular differences.

## Methods

### Data source

Data on breast cancer methylation and mRNA expression profiles were downloaded from the TCGA data portal (https://gdc-portal.nci.nih.gov/). A total of 1241 samples were available, 628 of which were marked as luminal type (type A or B). Luminal type samples with methylation data (Platform: Illumina Infinium Human Methylation 450) and mRNA-Seq data (Platform: Illumina HiSeq 2000 RNA sequencing) were selected for further analysis. From this analysis, 231 samples were obtained, including 21 control (non-cancerous) tissues and 210 luminal breast cancer tissues. Among the 210 breast cancer tissues, 191 had the corresponding survival information and status.

### Primary screening of methylation data

Continuous variables were expressed as mean ± standard deviation (SD), and categorical variables were expressed as sample size (composition ratio) in clinical information statistics. Methylation sites with significantly different methylation levels were obtained by comparing the methylation levels between cancer and control samples using the Wilcoxon rank sum test. The influence of the methylation level of these sites on the overall survival of luminal breast cancer patients was analysed using the Cox model. Sites with high correlation were further analysed using a linear correlation model to assess the relationship between their methylation levels and the corresponding mRNA expression. We obtained a subset of methylation sites, the methylation levels of which were significantly correlated with the corresponding mRNA expression. Lastly, the relationship between mRNA expression levels and luminal breast cancer prognosis was assessed using the Cox model. The resulting genes were considered as key genes in luminal breast cancer, as their methylation levels and mRNA expression levels were significantly correlated with each other and with the prognosis.

### Screening for significantly differentially expressed genes

Based on the cut-off methylation levels (mean methylation levels) of the key genes, samples were classified as hypo- or hypermethylated. Differences in expression were analysed by comparing mRNA levels of hypo- and hypermethylation groups with those of the control group (21 samples) using the EdgeR package (R3.1.0) [16]. False discovery rate (FDR) was calculated using the multtest R package. Genes with an FDR of < 0.05 and an expression fold change of > 1.5 or < 0.67 were considered to be significantly differentially expressed genes.

### Screening for prognosis-relevant signature and risk score calculation

Significantly differentially expressed gene mRNAs that significantly correlated with prognosis were screened using Cox regression in the survival R package [17]. *P-*value and the prognosis-relevant coefficient *β* were obtained using log-rank test. Risk score was defined as follows:

$$ \mathrm{Risk}\ \mathrm{score}={\beta}_{gene1}\times \mathit{\exp}r{}_{gene1}+{\beta}_{gene2}\times \mathit{\exp}{r}_{gene2}+\cdots +\beta {}_{gene n} \times \exp {r}_{gene n,} $$

Where *β* and *expr* are the prognosis-relevant coefficient and expression level of the corresponding gene, respectively. The risk score was calculated for each sample, and the median risk score was set as the cut-off for determining which samples were divided into low-and high-risk groups [18–20].

### Correlation analysis between samples and their clinical information

Clinical information of the corresponding samples, including age, ER status, HER2 status, progesterone receptor (PR) status, pathological stages (M, N and T), radiation therapy and the risk score, were integrated. To identify the clinical features significantly related to prognosis, a prognosis-relevant correlation analysis between the samples and their clinical information was performed using univariate and multivariable Cox regression in the survival R package. The resulting clinical features were analysed using Kaplan–Meier survival curves.

### Validation of the risk scoring system

To validate the risk scoring system, the expression profile under the accession number GSE22226 (platform GPL1708) [21] from the Gene Expression Omnibus (GEO) database (http://www.ncbi.nlm.nih.gov/gds/?term=) and the Metabric cohort dataset (http://www.cbioportal.org/study?id=brca_metabric#summary) were downloaded as independent validation datasets. In the GSE22226, a total of 130 breast cancer samples were included in the dataset, 57 of which were of luminal type with survival information and were used for validation. The expression levels of signature genes were extracted, and the risk score was calculated for each sample. The samples were divided into low- and high-risk groups, and the clinical information was also integrated, as described previously. Validation was performed by comparing the distribution of risk scores and overall survival time (in days) as well as by Kaplan–Meier survival curve analysis. In the Metabric cohort, a total of 1979 patients with good follow up data were included, 1140 of which were luminal disease. The risk score was calculated as described previously. Kaplan–Meier survival analysis based on risk score model system and Luminal subtypes were performed. Low- and high-risk groups were divided by signature genes in Luminal A and Luminal B samples from the Metabric cohort and the TCGA database. Prognosis value of low- and high-risk groups were shown by kaplan–Meier survival analysis.

### Screening of genes differentially expressed between low- and high-risk groups

**Fig. 1:**
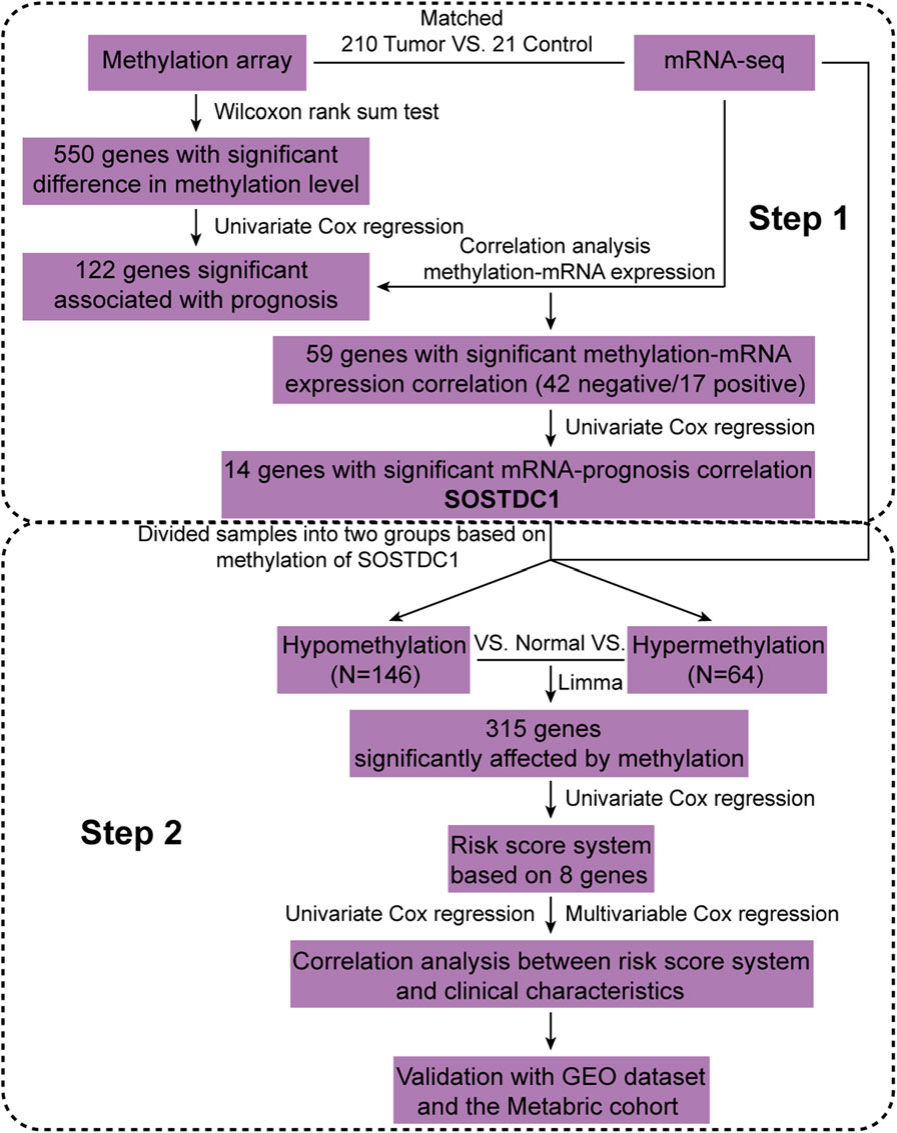
A flow chart showing the analysis process of this study

Using the TCGA dataset, the samples were divided into low- and high-risk groups according to their risk scores. Differences in gene expression levels between low- and high-risk groups were analysed using the limma R package, and FDR was calculated using the multtest R package. Genes with an FDR of < 0.05 were considered to be significantly differentially expressed. A correlation analysis of their expression levels and risk scores was performed, followed by two-way hierarchical clustering (shown as a heat map), Gene Ontology (GO) analysis and KEGG pathway analysis. The entire analysis process is shown in Fig. 1 as a flow chart.

## Results

### Features of the samples

A total of 210 luminal breast cancer samples and 21 control samples were included in our analysis, which were obtained from patients with a mean age of 60.73 ± 13.18 and 56.38 ± 14.59 years, respectively. Other clinical information, including ER status, can be found in the Supplementary materials (Additional file 1: Figure S1).

### Methylation sites associated with breast cancer prognosis

We compared the methylation levels of the cancer and control issues using the Wilcoxon rank sum test. A total of 550 methylation sites displayed significant methylation differences (Additional file 2: Figure S2 and Additional file 3: Figure S3). The relationship between methylation levels and breast cancer prognosis was analysed using the Cox proportional hazards model, and 122 methylation sites were found to be significantly associated with prognosis (Additional file 4: Figure S4).

Aberrant methylation is considered to be correlated with gene expression. We next evaluated the correlation between methylation levels and mRNA expression. For genes with methylation sites associated with prognosis, their mRNA expression values were extracted from the mRNA-Seq data. The correlation analysis of 122 methylation site–mRNA expression pairs revealed that 59 pairs were significantly correlated (*P* < 0.05), 42 of which were negatively correlated and 17 were positively correlated (Additional file 5: Figure S5). Further analysis of the correlation between mRNA expression levels of these genes and prognosis revealed that 14 of them, including *VIM*, *EPHX3*, *ACVR1*, *ANGPT1*, *TPM3*, *ALOX15*, *DIO1*, *KCNJ2*, *RSPH9*, *SOSTDC1*, *SYCP2*, *MACF1*, *TDRD5* and *CELSR3*, were significantly related to breast cancer prognosis (Table 1). As no previous reports showed the existence of the methylation sites of these genes in other types of breast cancer, we consider that the methylation sites of these genes are specific to luminal breast cancer.

**Table 1:** mRNAs significantly related to luminal breast cancer prognosis

Gene	Meth-ID	HR	lower.95	upper.95	*p*
VIM	cg12874092	0.077	0.011	0.566	0.0117
EPHX3	cg05488632	2.483	1.214	5.078	0.0127
ACVR1	cg16682903	1.120	0.675	1.351	0.0155
ANGPT1	cg09396217	2.472	1.116	5.474	0.0257
TPM3	cg24490338	0.126	0.019	0.842	0.0326
ALOX15	cg15843823	1.419	1.018	1.976	0.0387
DIO1	cg19526600	0.823	0.676	1.003	0.0435
KCNJ2	cg19042062	0.380	0.132	1.096	0.0435
RSPH9	cg01344171	1.481	0.962	2.282	0.0447
SOSTDC1	cg06363129	0.532	0.287	0.986	0.0449
SYCP2	cg07347645	1.628	0.969	2.737	0.0456
MACF1	cg22233974	2.244	0.864	5.825	0.0468
TDRD5	cg09656934	1.414	0.951	2.104	0.0473
CELSR3	cg06621358	0.618	0.376	1.017	0.0481

### Sample grouping based on methylation β-value of SOSTDC1

One identified gene, sclerostin domain-containing protein 1 (*SOSTDC1*) was of particular interest, because *SOSTDC1* showed a higher methylation level in breast cancer tissues than the other 13 genes (Additional file 6: Table S1) and among the three genes with the highest significant levels (*KCNJ2*, *CELSR3* and *SOSTDC1*), *SOSTDC1* was the only gene that has been reported to be associated with metastatic survival of breast cancer [22, 23], which indicates that *SOSTDC1* plays a complex role in metastatic breast cancer, so we chose *SOSTDC1* for further study.

Extracted data on *SOSTDC1* methylation for breast cancer and the control samples indicated that *SOSTDC1* methylation levels were significantly higher in cancer tissues than in control tissues (*P* = 3.05E-36) (Additional file 7: Table S2). The mean methylation *β*-value for breast cancer tissues was determined to be approximately 0.7 (indicated as a black line) and was set as the cut-off. Based on this cut-off value, cancer tissues were divided into hypo- and hypermethylation groups, which contained 64 and 146 samples, respectively.

### Screening of differentially expressed genes

mRNAs with low expression levels (expression value < 5) were removed from TCGA dataset, leaving a total of 11,858 mRNAs. The density peak of the expression levels significantly increased after removal of low-expressing mRNAs (Additional file 8: Table S3). We then compared control samples with both hypo- and hypermethylation samples using significant difference analysis. In total, 217 genes in the *SOSTDC1* hypomethylated group and 312 in the *SOSTDC1* hypermethylated group displayed significantly differential expression. We obtained 315 genes by combining the two groups.

### Identification of signature genes and construction of the risk scoring system

The survival information and status of 191 breast cancer tissues in the TCGA dataset were available and used for survival analysis. Among the differentially expressed genes, 67 were identified using Cox regression analysis, with their mRNA levels significantly related to prognosis (*P* < 0.05) (Additional file 9: Table S4).

To identify prognosis-associated signature genes, 67 genes were sorted according to their *P*-values derived from Cox regression log-rank test. Top n genes were used to construct a series of risk scoring systems (Table 2). The corresponding risk scores were calculated, and samples were divided into low- and high-risk groups under each risk scoring system. Their correlation with prognosis and the corresponding area under the curve (AUC) are shown in Table 3. The correlation between low- and high-risk groups and prognosis was at a maximum when the top seven genes were used, while the AUC [20] was maximized when the top eight genes were used. Therefore, we used the top eight genes as signature genes [establishment of cohesion 1 homolog 2 (*ESCO2*), Protein Kinase C And Casein Kinase Substrate In Neurons 1 (*PACSIN1*), cell division cycle associated 2 (*CDCA2*), polymeric immunoglobulin receptor (*PIGR*), pleiotrophin (*PTN*), repulsive guidance molecule A (*RGMA*), kallikrein-related peptidase 4 (*KLK4*) and centromere protein A (*CENPA*)].

**Table 2:** Prognosis-related genes of luminal breast cancer

Symbol	*P* value^a^	Hazard Ratio	β	*P* value^b^	AUC
ESCO2	0.00033	1.500	5.380	0.932	0.642
PACSIN1	0.00073	0.610	2.060	0.448	0.74
CDCA2	0.00094	2.310	−7.640	0.3137	0.781
PIGR	0.00107	0.404	−1.320	0.01191	0.878
PTN	0.00108	0.796	−2.600	0.00942	0.903
RGMA	0.0011	1.130	3.680	0.00235	0.914
KLK4	0.0011	0.359	−1.172	0.000251^c^	0.961
CENPA	0.00127	3.230	10.400	0.00128	0.993^d^
ADAMTS14	0.00156	1.180	−3.740	0.00237	0.992
ACAN	0.00262	0.553	1.663	0.00228	0.991

^a^Correlation of gene expression with overall survival

^b^Correlation of low- and high-risk groups divide by risk score with prognosis

^c^*P* value with the highest significance

^d^*P* value with highest AUC

**Table 3:** Univariate and multivariate Cox regression analysis of the relationship between clinical data and prognosis for TCGA dataset

Variable	Univariate Cox		Multivariable Cox	
	*p*-value	HR(CI)	*p*-value	HR(CI)
Age (58.02 ± 13.28 y)	0.163	1.045(0.982~ 1.11)	–	–
ER (Positive/Negative)	0.174	0.225(0.0263~ 1.929)	–	–
HER2 (Positive/Negative)	0.289	3.51(0.345~ 5.71)	–	–
pathologic_M(M0/M1)	0.586	1.564(0.331~ 2.806)	–	–
pathologic_N (N0/N1/N2/N3)	0.639	1.159(0.625~ 2.15)	–	–
pathologic_T (T1/T2/T3/T4)	0.0915	1.821(0.909~ 2.652)	–	–
Stage (I/II/III/IV/V)	0.121	1.586(0.885~ 2.841)	–	–
Radiation therapy (Yes/No)	0.184	0.336(0.0673~ 1.679)	–	–
Luminal type (A/B)	*0.03361* ^a^	1.698(1.036–2.782)	*0.0119* ^a^	2.508(1.225~ 5.134)
PR (Positive/Negative)	*0.00713* ^a^	0.125(0.0274~ 0.568)	0.4649	0.725(0.306~ 1.718)
Riskscore	*0.00115* ^a^	1.095(1.037~ 1.156)	*0.04382* ^a^	1.229(0.729~ 2.071)

^a^*P* values with significance

**Fig. 2:**
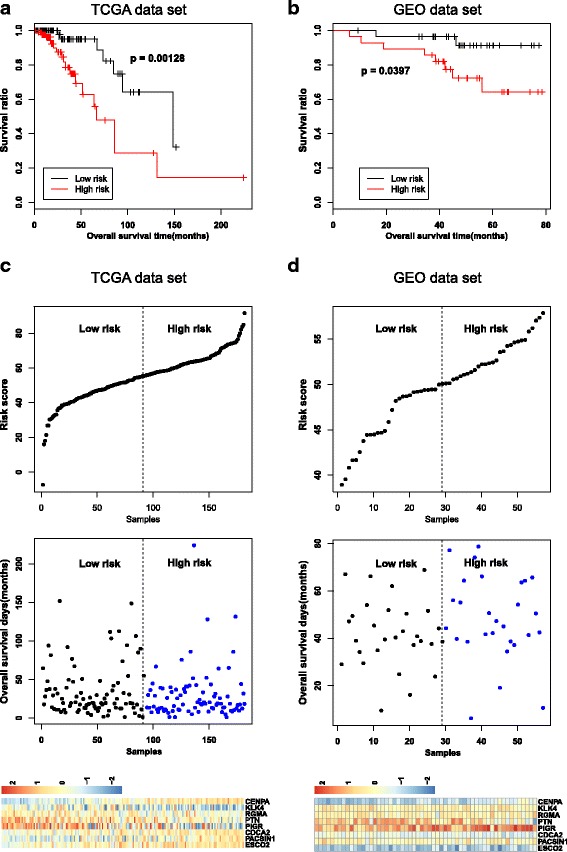
Comparison of prognosis, risk scores and expression patterns of signature genes. **a** and **b** Kaplan–Meier survival curves of the low- and high-risk groups between the TCGA and GEO samples. Survival curves of low- and high-risk groups are indicated as black and red lines, respectively. *P*-value indicates significance for the log-rank test. **c** and **d** Distribution of risk scores, overall survival time and expression profiles of signature genes in the TCGA and GEO samples. Expression profiles are shown as heatmaps

Risk scores were calculated using the risk scoring system containing the top eight genes (Additional file 10: Table S5). The samples were divided into low- and high-risk groups based on the median risk score (55.27). Kaplan–Meier survival curve analysis showed that sample grouping by this risk scoring system correlated significantly with prognosis (Fig. 2a). Moreover, the overall survival was significantly longer in the low-risk group than in the high-risk group (*P* = 0.00128).

**Fig. 3:**
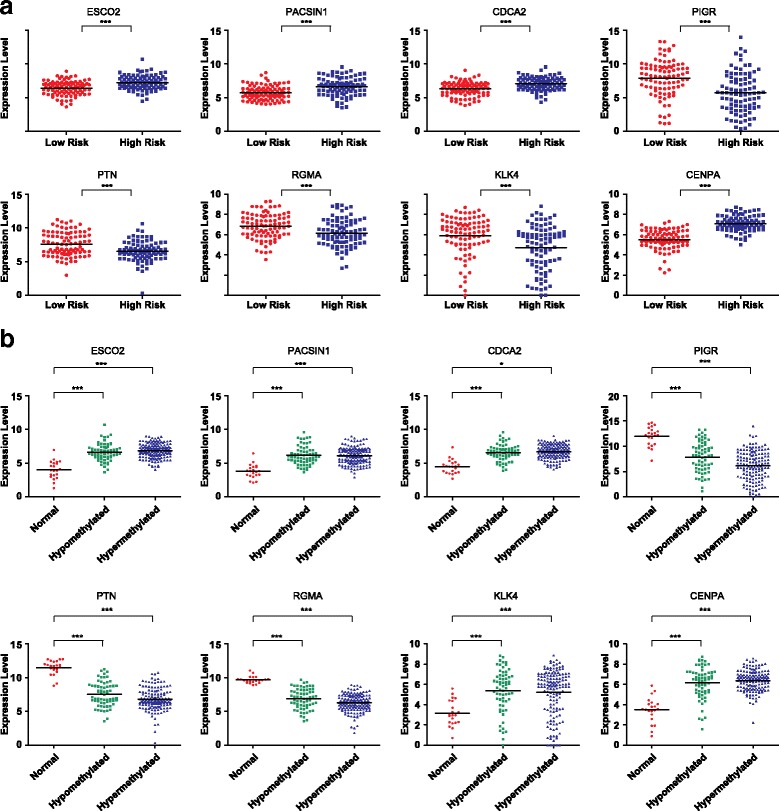
Difference in expression of signature genes. **a** Comparison of expression between the low- and high-risk groups. **b** Comparison of expression among the normal, hypomethylated and hypermethylated groups. ‘***’ indicates *P*-value < 0.001

**Fig. 4:**
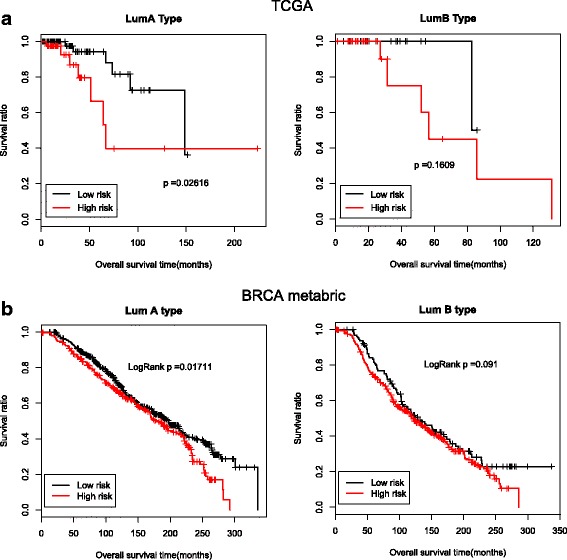
Prognosis of low- and high-risk groups in Luminal A and Luminal B samples from the TCGA and the Metabric cohort dataset. **a** Kaplan–Meier survival curves of low- and high-risk groups divided by eight signature genes in Luminal A and Luminal B samples from the TCGA database, respectively. The black line indicates the low-risk group, and the red line indicates the high-risk group. **b** Kaplan–Meier survival curves of low- and high-risk groups divided by eight signature genes in Luminal A and Luminal B samples from the Metabric cohort, respectively. The black line indicates the low-risk group, and the red line indicates the high-risk group

We examined the expression levels of the signature genes, which revealed that their expression levels significantly differed between the low- and high-risk groups (Fig. 3a, Additional file 11: Table S6). Significant differences in expression levels were also found between the control samples and the hypo-or hypermethylation groups. (Figure 3b, Additional file 12: Table S7). However, no significant differences were found between the hypo and hypermethylation groups. Moreover, low- and high- risk groups of Luminal A samples divided by the signature genes significantly correlated with the survival ratio. As to Luminal B samples, the survival rate of low risk group was also higher than that of high risk group, although this correlation was not so significant (Fig. 4a). We also evaluated if the prediction analysis of microarray 50 (PAM50) intrinsic subtypes, which are prognostic independent of standard clinicopathologic factors, could well differentiate Luminal A and Luminal B subtypes. As shown in Additional file 13: Table S8, PAM50 was not the key signature genes for splitting Luminal A and Luminal B subtypes in the patient cohort of this study (Additional file 13: Table S8).

To further validate the signature genes, we used the Metabric cohort [6]. The Metabric cohort included 1979 patients with good follow up data of which 1140 were of luminal disease. These luminal samples were also divided into low- and high-risk groups by median risk score based on the signature genes. Low- and high- risk groups of Luminal A samples significantly correlated with the survival ratio. As to Luminal B samples, this correlation also showed no significant difference (Fig. 4b). We combined Luminal A and Luminal B data of the Metabric cohort and made a table (2 × 2) with Luminal A, Luminal B, high score, low score. We found that the distribution of Luminal A and Luminal B samples between low- and high groups were significantly different. Most Luminal A samples fell in the low score group and the majority of Luminal B samples fell in the high score group (chisq test, X-squared = 137.0685, *p* < 2.2e-16) (Table 4). Kaplan–Meier analysis revealed that high score group was significantly correlated with poor survival of patients with luminal breast cancer in Metabric cohort (Additional file 14: Table S9). The classification of Luminal A and Luminal B also significantly correlated with survival ratio of luminal subtype patients. The patients with Luminal A breast cancer had a longer survival time than the patients with Luminal B breast cancer (Additional file 14: Table S9).

**Table 4:** Fourfold table showing the number of Luminal A and Luminal B samples in low- and high groups

	Luminal A	Luminal B	*p* value
High group	242	328	2.20E-16
Low group	437	133	

### Validation of the risk scoring system

The GSE22226 expression profile dataset was used for validating our risk scoring system. The expression level values of the signature genes were extracted and the risk score for each sample was calculated (Additional file 15: Table S10). Each sample was divided between the low-risk group (29 samples) and high-risk group (28 samples). The survival ratio in both groups was evaluated. Similar to the results seen in the TCGA training dataset, the survival ratio in the low-risk group was significantly higher than that of high-risk group (*P* = 0.0397) in the validation dataset (Fig. 2b). Additionally, the distribution of the risk scores and overall survival time were similar between the validation and training datasets (Fig. 2c and d). All validation results that we obtained confirmed the robustness and reliability of our risk scoring system.

### Correlation between clinical features and prognosis

**Fig. 5:**
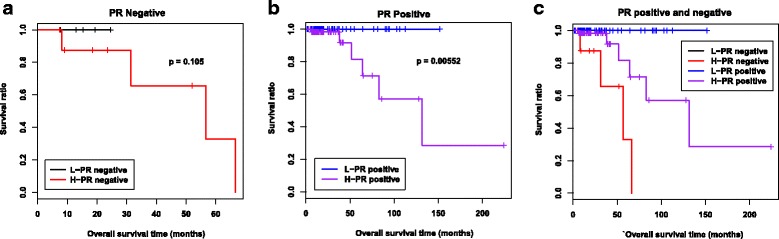
Prognosis of the low- and high-risk groups in PR-negative and PR-positive samples. **a** Kaplan–Meier survival curves of the low- and high-risk groups in PR-negative samples. The black line indicates the low-risk group, and the red line indicates the high-risk group. *P*-value indicates the significance of the difference between the two groups. **b** Kaplan–Meier survival curves of the low- and high-risk groups in PR-positive samples. The blue line indicates the low-risk group, and the violet line indicates the high-risk group. *P*-value indicates the significance of the difference between the two groups. **c** Combination of the Kaplan–Meier survival curves from (**a**) and (**b**)

Clinical information was integrated for prognosis-related correlation analysis (Additional file 16: Table S11). Univariate Cox regression indicated that both the PR status and the risk score were significantly correlated with prognosis, whereas multivariable Cox regression indicated that only the risk score was an independent prognostic factor (Table 3). Further analysis demonstrated that the survival ratio was higher in the low-risk group than in the high-risk group in both PR-positive (PR+) and PR-negative (PR−) patients (Fig. 5; 14 samples vs. 123 samples; *P* = 0.105 vs. *P* = 0.00552). However, the scoring system that we used was more sensitive in PR+ than in PR− samples because of the difference in *P*-values.

### Gene expression differences in the different groups

**Fig. 6:**
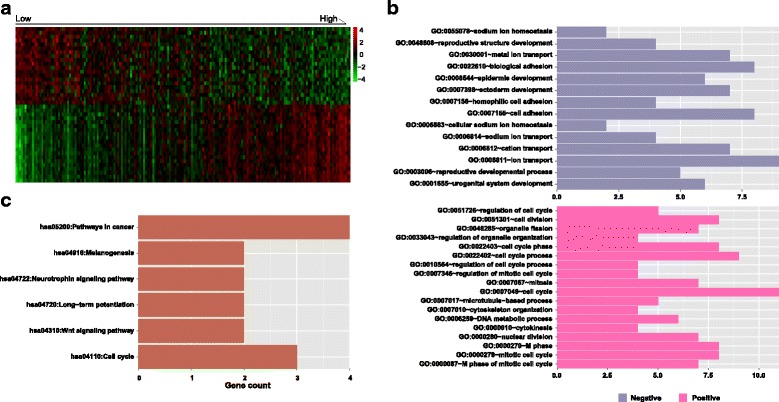
Functional annotation of genes differentially expressed between the low- and high-risk groups. **a** Hierarchical clustering analysis of the expression levels of the top 20 positively and negatively related genes. **b** GO analysis of negatively (upper) and positively (lower) related genes. **c** KEGG pathway analysis of significantly correlated genes

We found 121 genes that exhibited significant differential expression (FDR < 0.05) between the low- and high-risk groups. The correlation analysis indicated that 88 of them were positively correlated and 33 were negatively correlated with the risk score (Additional file 17: Table S12). The expression patterns of the top 20 positively and negatively correlated genes are shown as heatmaps using hierarchical clustering analysis in Fig. 6a. Further biological function enrichment analysis revealed that most positively correlated genes were involved in the cell cycle, whereas most negatively correlated genes were involved in development, cell adhesion, ion transport and homeostasis (Fig. 6b, Additional file 18: Table S13). KEGG pathway analysis indicated that these genes were correlated with cancer, cell cycle and signalling pathways (Fig. 6c, Additional file 19: Table S14). The overall results of the KEGG pathway analysis were consistent with those of the biological function enrichment analysis, considering the complex relationship between tumourigenesis and multiple biological processes, such as cell cycle, cell adhesion and development.

## Discussion

Variations in methylation profiles are of considerable importance in breast cancer onset and progression [4]. Methylation profiles differ among breast cancer subtypes and may influence gene expression [10]. In the present study, we focused on luminal breast cancer. We downloaded the data from TCGA, a public database that catalogues the genetic profiles of over 30 human tumors, including breast cancer. This platform contains many types of data, such as gene expression, exon expression, miRNA expression, copy number variation (CNV), single nucleotide polymorphism (SNP), mutations, DNA methylation, and protein expression. However, the TCGA database has poor follow up data. A majority of the samples are concatenated shortly after diagnosis, which limited the number of available samples in our study. Due to poor follow up data, the TCGA patient material is not representative of any real breast cancer population. Using data from the TCGA, we identified a set of prognosis-related methylation sites and further evaluated their relationship with corresponding mRNA expression. We identified 14 genes (Table 2) whose mRNA expression levels, methylation levels and prognosis of breast cancer were significantly correlated.

Among these genes, *SOSTDC1* is of special interest, considering its complex role and potential importance in metastatic breast cancer. *SOSTDC1* is a member of the sclerostin gene family and is actively involved in the bone morphogenic protein and Wnt signalling pathways. *SOSTDC1* mRNA levels are downregulated in breast cancer and are associated with survival [22, 23]. The elevation in *SOSTDC1* methylation level in tumour tissues (Additional file 7: Table S2) may explain *SOSTDC1* downregulation in breast cancer because promoter methylation has an inhibitory effect on gene expression. Because *SOSTDC1* is closely associated with luminal breast cancer, we divided the samples into hypo- and hypermethylation groups based on *SOSTDC1* methylation levels. Another DNA methylation signature, *SAM40*, was reported to discriminate patients with luminal A breast cancer between good prognoses and poor prognoses [24]. This highlights the feasibility of the sub-classification of the patient groups based on DNA methylation signature. Future studies might focus on the combination of *SAM40* and *SOSTDC1* in the prognostic prediction of luminal breast cancer.

To identify signature genes in luminal breast cancer, we also compared mRNA expression profiles between breast cancer and control tissues. A total of 67 differentially expressed genes were found to be significantly correlated with prognosis. Further analysis identified eight signature genes (*ESCO2*, *PACSIN1*, *CDCA2*, *PIGR*, *PTN*, *RGMA*, *KLK4* and *CENPA*). These signature genes were used to construct a prognosis-related risk scoring system, based on which samples were classified into low-and high-risk groups. The luminal breast cancer samples from the TCGA and the Metabric cohort were used to validate this system. Interestingly, we found prognostic differences within the Luminal A breast cancer patients in both databases, although the two lines in Fig. 4b were almost overlapping. No significant prognostic differences were found within Luminal B samples, indicating that this risk score system might have prognostic value for patients with Luminal A breast cancer.

Many research groups have focused on the prediction of prognosis and chemotherapeutic benefits by construction of a risk system based on gene expression profile, such as the 70-gene predictor [25] and the 50-gene signature [26]. The 50-gene signature test (PAM50) is one of the most widely accepted systems for the prediction of clinical outcomes in women with distinct intrinsic subtypes [26]. In the patient cohorts of this analysis, our signature genes were more suitable for splitting Luminal A and Luminal B subtypes than PAM50. However, a limitation of our study is that the cohort of luminal breast cancer samples in TCGA was small. Future studies will utilize larger patient cohorts and enrich the clinical data to validate our risk system.

Previous studies have shown that most signature genes are involved in cancer progression, even though they may not be directly involved in breast cancer. It has been reported that *ESCO2*, *CDCA2* and *CENPA* are cell cycle-related genes involved in cancer progression. *ESCO2* is an acetyltransferase, which is required for cohesion acetylation and the establishment of sister chromatid cohesion in the S phase [27, 28], and has been found to be upregulated in melanoma [29]. *CDCA2* is required in the formation of mitotic chromatin and is involved in the progression of human squamous cell carcinoma [30]. *CENPA* is essential for centromere integrity and chromosome segregation, and *CENPA* dysregulation may promote tumourigenesis due to the resulting genome instability [31–33]. Other signature genes, including *PTN*, *KLK4*, *RGMA* and *PIGR*, have also been reported to be involved in cancer progression. Increased *PTN* [34, 35] and *KLK4* [36–38] expression is strongly associated with the progression of different malignant cancers. Decreased *PIGR* expression has been found in colon tumours [39], while *RGMA* has been reported to have an inhibitory effect on cancer progression [40, 41]. The remaining signature gene, *PACSIN1*, is important in endocytosis and synaptic vesicle recycling [42, 43]. Although its direct involvement in cancer has not been reported, it may play an indirect role in cancer progression.

Our results also demonstrated significant differences in the expression of these signature genes between low- and high-risk groups and between the control and cancerous tissues (Fig. 3). Our GO and pathway analyses revealed that the genes that were expressed differentially between the low- and high-risk groups were mainly involved in biological processes, such as cell cycle and cancer progression (Fig. 5b and c).

There are limitations in our manuscript. The gene signature is derived from the segregation of patients based on methylation level of only one gene (SOSTDC1), which could cause bias of data analysis. The eight-gene signature was screened based on bioinformatics analysis and this study may just provide clues for future study of patients with luminal breast cancer. The future focus of our work is to collect more samples and improve our risk score system experimentally.

Taken together, our results supported the role of these genes, consistent with their biological functions, in the development and progression of luminal breast cancer.

## Conclusions

In conclusion, we identified 14 genes that were closely related to luminal breast cancer prognosis. Their methylation levels, mRNA expression and prognosis were significantly correlated with each other. We also identified eight clinically valuable signature genes for luminal breast cancer, and a risk scoring system was built upon this profile. Our results demonstrated that this system is stable and effective in predicting prognosis and can be used in the clinical diagnosis and treatment of luminal breast cancer. Further functional studies on the signature genes are needed to gain a deeper insight into the roles they play in breast cancer onset, with specific emphasis on *PACSIN1* because its direct involvement in cancer has not yet been reported.

## Additional files

Additional file 1: **Figure S1.** Box plot showing the methylation levels of 14 genes in the control and luminal breast cancer tissues. Blue and orange boxes indicate methylation levels in the control and cancer samples, respectively. (PDF 20 kb)

Additional file 2: **Figure S2.** Distribution of *SOSTDC1* methylation levels in the control and luminal breast cancer tissues. Red and blue points indicate methylation levels in the control and cancer samples, respectively. Black lines indicate the mean methylation levels of the corresponding samples. (PDF 19 kb)

Additional file 3: **Figure S3.** Distribution of mRNA expression density. Solid and dashed lines indicate the density distribution curve before and after the removal of mRNA with low expression levels, respectively. (PDF 7 kb)

Additional file 4: **Figure S4.** Kaplan–Meier survival curves of low- and high-risk groups divided by PAM50 in Luminal A and Luminal B samples, respectively. The black line indicates the low-risk group, and the red line indicates the high-risk group. (TIFF 772 kb)

Additional file 5: **Figure S5.** Kaplan–Meier survival analysis based on risk score model system (a) and Luminal subtypes using the Metabric cohort (b). (a) The black and red lines indicates the low-risk group and the high-risk group; (b) The black and red lines indicates the Luminal A and Luminal B breast cancer tissues. (PDF 29 kb)

Additional file 6: **Table S1.** Clinical information of 210 luminal breast cancer samples. (XLSX 10 kb)

Additional file 7: **Table S2.** List of DEmethylated sites. (XLS 105 kb)

Additional file 8: **Table S3.** Intersection of DEGs in hypo and hyper methylated groups. (XLS 96 kb)

Additional file 9: **Table S4.** Methylation sites that significantly associated with breast cancer prognosis. (XLS 38 kb)

Additional file 10: **Table S5.** Correlated methylation site–mRNA expression pairs. (XLS 24 kb)

Additional file 11: **Table S6.** Survival prognostis related genes from cox regression. (XLS 22 kb)

Additional file 12: **Table S7.** Clinic and gene factor information for model. (XLS 62 kb)

Additional file 13: **Table S8.** Model gene factor expression in Low and High risk samples. (XLS 52 kb)

Additional file 14: **Table S9.** Model gene factor expression in Normal and Cancer samples. (XLS 57 kb)

Additional file 15: **Table S10.** Clinic and gene expression information for testing dataset GSE22226. (XLS 32 kb)

Additional file 16: **Table S11.** Clinic information for samples in this study. (XLS 56 kb)

Additional file 17: **Table S12.** DEGs screened from High and low risk groups. (XLS 31 kb)

Additional file 18: **Table S13–1.** Biology process annotations for DEGs negatively correlated to High and Low risk. **Table S13–2.** Biology process annotations for DEGs positively correlated to High and Low risk. (XLS 23 kb)

Additional file 19: **Table S14.** KEGG pathways annotations for DEGs from High and Low risk. (XLSX 10 kb)

## Abbreviations

AUC: area under the curve
*CDCA2*: cell division cycle associated 2
*CENPA*: centromere protein A
CNV: copy number variation
CpG: Cytosine-Phosphate-Guanine
*ESCO2*: establishment of cohesion 1 homolog 2
GEO: Gene Expression Omnibus
GO: Gene Ontology
*GSTP1*: Glutathione S-transferase Pi 1
*KLK4*: kallikrein-related peptidase 4
*PACSIN1*: Protein Kinase C And Casein Kinase Substrate In Neurons 1
PAM50: the prediction analysis of microarray 50
*PIGR*: polymeric immunoglobulin receptor
PR: progesterone receptor
*PTN*: pleiotrophin
*RASSF1*: Ras Association Domain family 1
*RGMA*: repulsive guidance molecule A
SD: standard deviation
SNP: single nucleotide polymorphism
*SOSTDC1*: sclerostin domain-containing protein 1
TCGA: The Cancer Genome Atlas
