# Supplementary figures and images for: Identification of methylation sites and signature genes with prognostic value for luminal breast cancer
Source: BMC Cancer. 2018 Apr 11;18:405. doi: 10.1186/s12885-018-4314-9 (PMC5896050; doi:10.1186/s12885-018-4314-9)

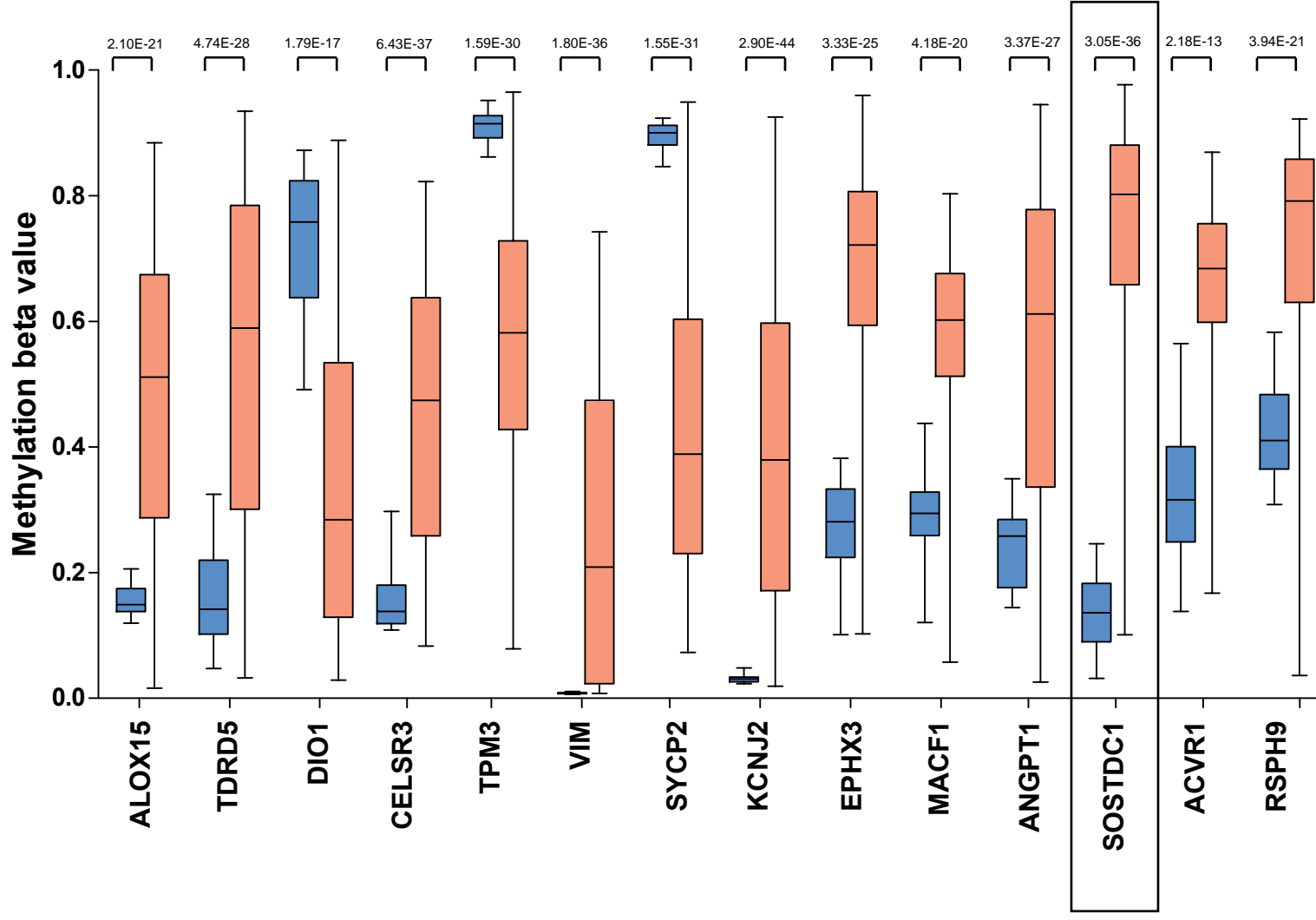

Supplement: Supplementary file 1 — Figure S1. Box plot showing the methylation levels of 14 genes in the control and luminal breast cancer tissues. Blue and orange boxes indicate methylation levels in the control and cancer samples, respectively. (PDF 20 kb) [file 12885_2018_4314_MOESM1_ESM.pdf]

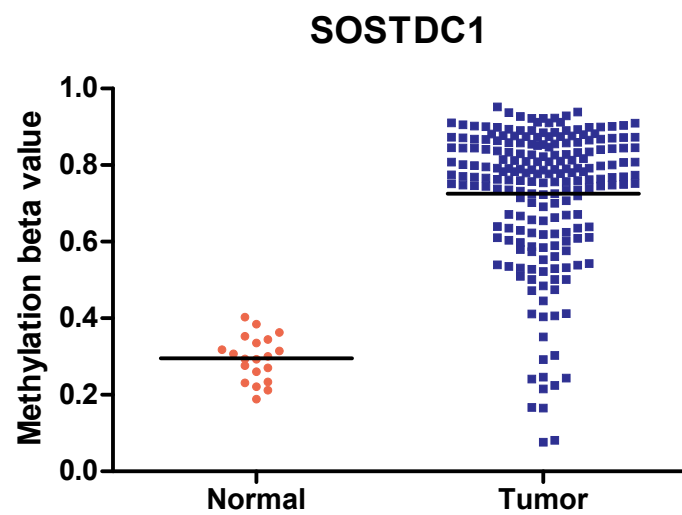

Supplement: Supplementary file 2 — Figure S2. Distribution of SOSTDC1 methylation levels in the control and luminal breast cancer tissues. Red and blue points indicate methylation levels in the control and cancer samples, respectively. Black lines indicate the mean methylation levels of the corresponding samples. (PDF 19 kb) [file 12885_2018_4314_MOESM2_ESM.pdf]

## Expression density distribution

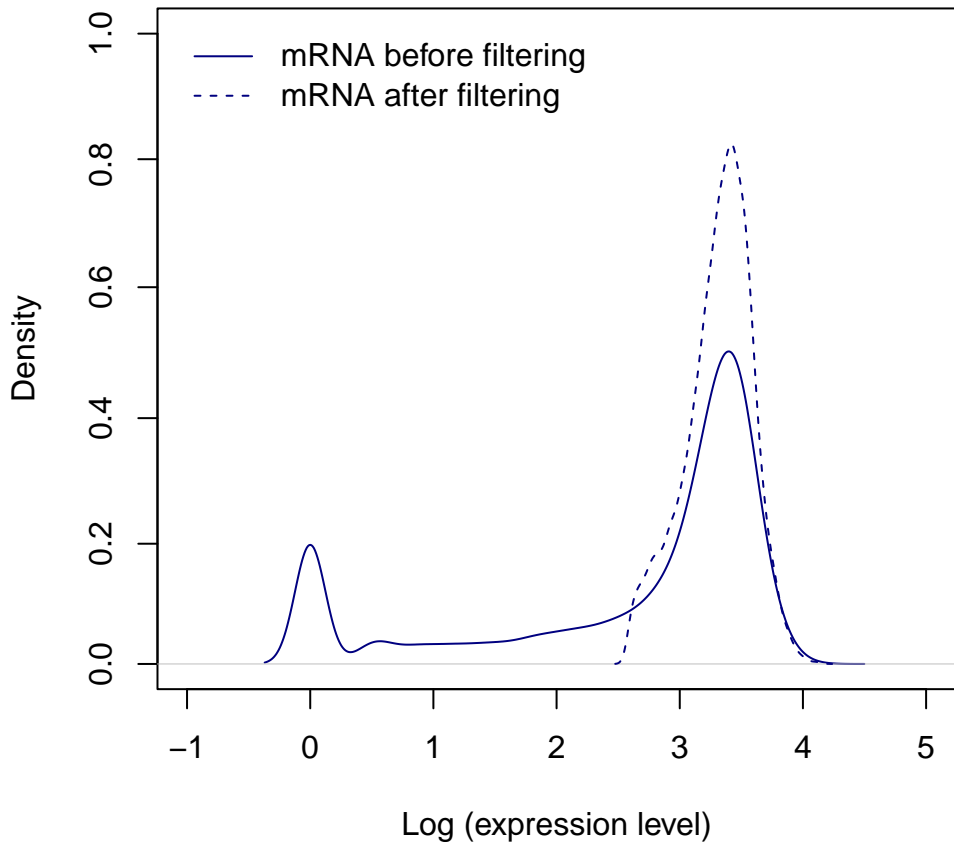

Supplement: Supplementary file 3 — Figure S3. Distribution of mRNA expression density. Solid and dashed lines indicate the density distribution curve before and after the removal of mRNA with low expression levels, respectively. (PDF 7 kb) [file 12885_2018_4314_MOESM3_ESM.pdf]

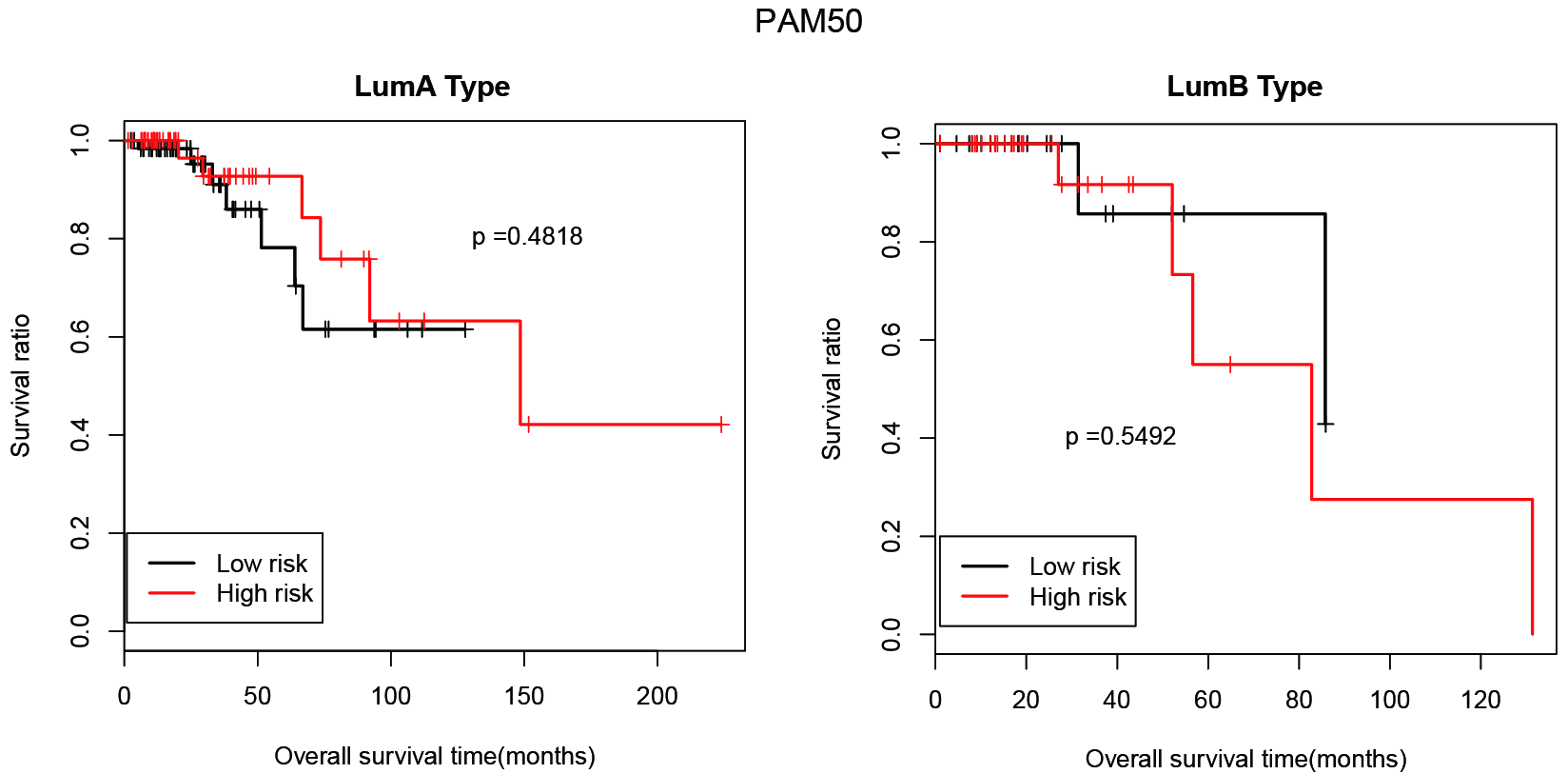

Supplement: Supplementary file 4 — Figure S4. Kaplan–Meier survival curves of low- and high-risk groups divided by PAM50 in Luminal A and Luminal B samples, respectively. The black line indicates the low-risk group, and the red line indicates the high-risk group. (TIFF 772 kb) [file 12885_2018_4314_MOESM4_ESM.tif]

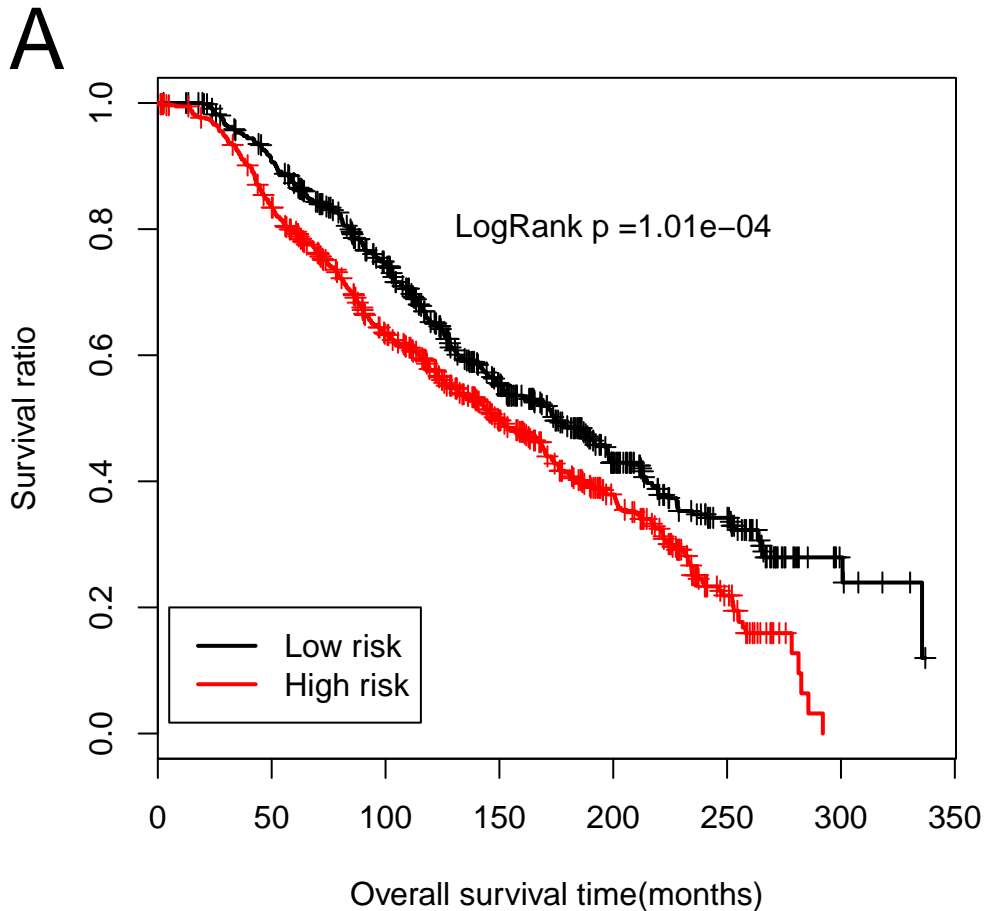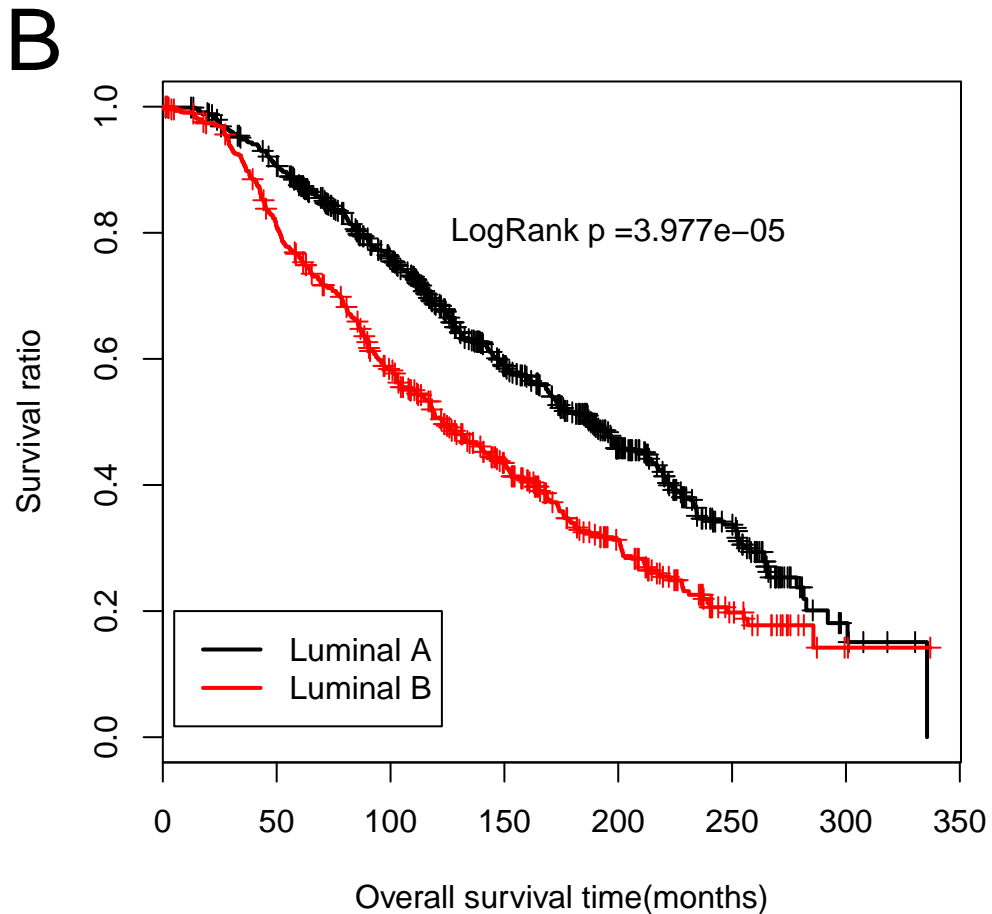

Supplement: Supplementary file 5 — Figure S5. Kaplan–Meier survival analysis based on risk score model system (a) and Luminal subtypes using the Metabric cohort (b). (a) The black and red lines indicates the low-risk group and the high-risk group; (b) The black and red lines indicates the Luminal A and Luminal B breast cancer tissues. (PDF 29 kb) [file 12885_2018_4314_MOESM5_ESM.pdf]
